# Inflammatory indexes as prognostic biomarkers in advanced triple negative breast cancer patients

**DOI:** 10.3389/fonc.2026.1876224

**Published:** 2026-07-15

**Authors:** Caterina Gianni, Emanuela Scarpi, Eva Blondeaux, Michela Palleschi, Filippo Merloni, Lorenzo Gasperoni, Fabio Puglisi, Elena Collovà, Palma Pugliese, Francesco Cognetti, Irene Giannubilo, Tommaso Ruelle, Lucia Del Mastro, Claudia Bighin, Antonino Musolino, Ugo De Giorgi

**Affiliations:** 1 Department of Clinical and Experimental Oncology and Hematology, Medical Oncology and Breast Unit, Istituto di Ricovero e Cura a Carattere Scientifico (IRCCS) Istituto Romagnolo per lo Studio dei Tumori (IRST) “Dino Amadori,”, Meldola, Italy; 2 Unit of Biostatistics and Clinical Trials, Istituto di Ricovero e Cura a Carattere Scientifico (IRCCS) Istituto Romagnolo per lo Studio dei Tumori (IRST) “Dino Amadori,”, Meldola, Italy; 3 U.O. Epidemiologia Clinica, Istituto di Ricovero e Cura a Carattere Scientifico (IRCCS) Azienda Ospedaliera Metropolitana, Genova, Italy; 4 Pharmaceutical Department, Unità Sanitaria Locale (USL) Toscana Centro, Prato, Italy; 5 Department of Medicine (DMED), University of Udine, Udine, Italy; 6 Department of Medical Oncology, Centro di Riferimento Oncologico (CRO) Aviano, National Cancer Institute, Istituto di Ricovero e Cura a Carattere Scientifico (IRCCS), Aviano, Italy; 7 Oncology Unit, Cancer Center Department, Azienda Socio Sanitaria Territoriale (ASST) Ovest Milanese, Legnano, Italy; 8 Unit of Oncology, Department of Medical, Azienda Socio Sanitaria Territoriale (ASST) Lariana, Como, Italy; 9 Department of Medical Oncology, Unicamillus International University, Rome, Italy; 10 Oncologia Medica, Istituto di Ricovero e Cura a Carattere Scientifico (IRCCS) Azienda Ospedaliera Metropolitana, Genova, Italy; 11 Department of Internal Medicine and Medical Specialties (DiMI), School of Medicine, University of Genova, Genova, Italy; 12 Department of Experimental Medicine, University of Salento, Lecce, Italy

**Keywords:** biomarkers, monocyte-to-lymphocyte ratio, neutrophil-to-lymphocyte ratio, prognostic, triple negative breast cancer

## Abstract

**Background:**

The immune system is known to be involved in the microenvironment of triple negative breast cancer (TNBC). Immune ratios such as the neutrophil-to-lymphocyte ratio (NLR), the platelet-to-lymphocyte ratio (PLR), the monocyte-to-lymphocyte ratio (MLR), and the systemic immune-inflammation index (SII) may reflect the functional status of the immune system in these patients and the involvement of circulating immune cells in cancer progression.

**Methods:**

We conducted a retrospective-prospective, observational multicenter analysis to investigate the association between inflammatory indexes (NLR, PLR, MLR, and SII), clinical characteristics and survival outcomes in patients with metastatic TNBC in the first-line setting.

**Results:**

Data from 114 consecutive patients with a diagnosis of metastatic TNBC were evaluated. At a median follow-up of 28 months, median PFS in the overall patient population was 8.6 months (95% CI 6.5–9.6), while median OS was 17.7 months (95% CI 13.7–23.9). All high inflammation-based scores evaluated at the diagnosis of metastatic disease were significantly associated with lower PFS, particularly high NLR (≥3), high MLR (≥0.34), high PLR (≥210), and high SII (≥836) (p <0.0001, p <0.0001, p = 0.0002, p <0.0001, respectively). Similarly, all indexes appeared to be significantly associated with lower OS, particularly NLR (≥3), SII (≥836), PLR (≥210), and MLR (≥0.34) (p <0.0001, p = 0.001, p = 0.002, and p = 0.0006, respectively). In multivariable analysis for predictors of OS, the number of metastatic sites, NLR, SII, and MLR remained significant.

**Conclusions:**

NLR, PLR, SII, and MLR are associated with PFS and OS in metastatic TNBC. Although our results require validation in larger prospective studies and contemporary cohorts treated with chemoimmunotherapy and novel agents, inflammatory ratios may represent feasible prognostic biomarkers in metastatic TNBC.

## Introduction

Triple-negative breast cancer (TNBC) accounts for almost 15% of all breast cancer (BC) diagnoses (1). It is known to be characterized by a worse prognosis and greater aggressiveness than other BC subtypes (2, 3).

The identification of treatment selection biomarkers for advanced TNBC patients remains an unmet need. Historically, treatment for TNBC has been exclusively represented by chemotherapy, with limited availability of targeted therapies or novel agents (4). This is partially attributable to the shortage of actionable biomarkers, such as the absence of hormonal receptor expression (defined as estrogen and progesterone receptor expression <10%) or the lack of efficacy of anti-human epidermal growth factor receptor 2 (HER2) therapies. However, this type of breast tumor is not a single entity. With advances in transcriptomic studies, the heterogeneity of TNBC has been further characterized into different molecular clusters, including the basal-like subtype with a highly proliferative phenotype (5–8), the immunomodulatory subtype expressing genes involved in antigen processing and presentation and immune cell and cytokine signaling pathways, the mesenchymal and mesenchymal stem-like subtypes displaying expression profiles related to cell motility, differentiation and epithelial–mesenchymal transition, and the luminal androgen receptor (LAR) subtype (4).

The immune microenvironment is highly involved in cancer promotion and progression. In particular, TNBC is known to be characterized by an increased number of tumor-infiltrating lymphocytes (TILs), which have been shown to have prognostic and potentially predictive value (5–8). High density of tumor associated macrophages (TAMs) in TNBC is associated with poor prognosis and indicates a higher risk of metastasis due to their immunosuppressive activity (9). Bianchini et al. reported that spatial data on the interactions among specific cells in the tumor microenvironment might be very informative regarding the benefit of adding an immune checkpoint inhibitor such as atezolizumab to chemotherapy (10).

A strong interplay between tissue immune cells and circulating inflammatory cells has been described. Immune ratios such as the neutrophil-to-lymphocyte ratio (NLR), the platelet-to-lymphocyte ratio (PLR), the monocyte-to-lymphocyte ratio (MLR) and the systemic immune-inflammation index (SII) may reflect the functional status of the immune system in these patients and the involvement of circulating immune cells in cancer progression. In particular, MLR was able to predict overall survival (OS) in advanced TNBC and contributed to the migration of circulating tumor cells (11, 12).

We conducted two retrospective/prospective observational studies including patients with metastatic BC with the aim of describing the natural history of the disease and capturing treatment strategies and outcome characteristics: the GIM14/BIOMETA study (ClinicalTrials.gov identifier: NCT02284581) and the IRST B114 study.

Our retrospective analysis, involving a homogeneous cohort of patients with metastatic TNBC enrolled in the two studies, contributes to enlarging the body of evidence regarding the role of inflammatory ratios as prognostic biomarkers in breast cancer.

## Methods

This is a retrospective analysis of a large cohort of consecutive patients with TNBC conducted within the GIM14/BIOMETA study (ClinicalTrials.gov identifier: NCT02284581), a retrospective/prospective multicenter observational study of the Gruppo Italiano Mammella (GIM) Study Group regarding treatment patterns and outcomes of patients with metastatic BC, and the IRST B114 study, a retrospective/prospective monocentric study of patients with breast cancer treated at IRCCS Istituto Romagnolo per lo Studio dei Tumori (IRST) “Dino Amadori.” The analysis was performed to investigate the association between inflammatory indexes (NLR, MLR, PLR, and SII), measured at baseline and at progression, and clinical characteristics and survival outcomes in patients with metastatic TNBC in the first-line setting. Data from patients diagnosed with metastatic TNBC between 2015 and 2021 were collected from the two studies.

Eligibility criteria for the present analysis were: 1) age ≥18 years; 2) diagnosis of unresectable, locally recurrent, or metastatic TNBC, defined by ER <10% and PR <10% on immunohistochemistry (IHC) analysis and a HER IHC score of 0, 1+, or +2 with negative *in situ* hybridization locally assessed on metastatic tumor tissue or primary tumor tissue when biopsy on metastatic sites was not performed, 3) availability of pretreatment peripheral blood neutrophil, lymphocyte, monocyte, and platelet counts. Pretreatment blood counts were defined as the closest available complete blood count obtained at the time of metastatic diagnosis before initiation of first-line systemic treatment for advanced or metastatic disease.

The data collected included leukocyte subsets (differentials) and clinical characteristics, including estrogen receptor (ER), progesterone receptor (PR), HER2 status, tumor dimension, grade, histological type, treatment administered, radiological assessment, metastatic sites categorized as visceral disease or non-visceral disease (visceral involvement was defined as liver, lung, and any other site except the lymph nodes, bone, skin/serosa, and brain), and clinical outcomes, including disease progression (PD) after first-line therapy. For a subset of patients, peripheral blood neutrophil, lymphocyte, and platelet counts at first progression were also collected.

We calculated the following ratios: the neutrophil-to-lymphocyte ratio (NLR; the ratio of neutrophil to lymphocyte counts), the platelet-to-lymphocyte ratio (PLR; the ratio of platelet to lymphocyte counts), the monocyte-to-lymphocyte ratio (MLR; the ratio of monocyte to lymphocyte counts), and the systemic immune-inflammation index (SII; neutrophil × platelet/lymphocyte count).

Cut-off values for inflammatory indexes were pre-specified before the present analysis and were derived from a previously published study by our group evaluating the prognostic role of circulating inflammatory indexes in metastatic breast cancer (11). The use of these previously established thresholds was intended to assess their reproducibility and prognostic relevance in an independent advanced/metastatic TNBC cohort while avoiding the derivation of data-driven cut-offs from the current dataset. The pre-specified cut-off values for NLR, PLR, SII, and MLR were 3, 210, 836, and 0.34, respectively.

### Statistical analysis

Progression-free survival (PFS) was defined as the time from initiation of first-line systemic treatment for advanced/metastatic TNBC to first documented disease progression or death from any cause, whichever occurred first. Patients who had not progressed at database lock were censored on the date of their last tumor assessment. Disease progression was assessed according to routine clinical and radiological evaluations at each participating center. No central radiological review was performed, in line with the real-world observational design of the study. Overall survival (OS) was defined as the time between the date of initiation of first-line systemic treatment for advanced/metastatic TNBC and the date of death from any cause or the date of the last follow-up visit.

Categorical variables were summarized using frequencies and percentages, whereas continuous variables were described using the median, minimum, maximum, and interquartile range (IQR). Survival curves were estimated using the Kaplan–Meier method and compared with the log-rank test. Univariable and multivariable Cox proportional hazards regression models were used to investigate potential predictors of PFS and OS and to estimate hazard ratios (HRs) with their 95% confidence intervals (CIs).

Given the biological and mathematical correlation among NLR, PLR, MLR, and SII, each inflammatory index was evaluated in a separate multivariable Cox regression model to avoid collinearity and improve model interpretability. Each model was adjusted for age, visceral involvement, and the number of metastatic sites before first-line treatment. The proportional hazards assumption was evaluated for each covariate by including an interaction term between the covariate and the logarithm of follow-up time. A non-significant interaction term (p >0.05) was considered indicative of the proportional hazards assumption.

No imputation of missing data was performed; analyses involving variables with missing values were conducted using available cases only. All p-values were two-sided, and p <0.05 was considered statistically significant. Statistical analyses were performed with SAS 9.4 software (SAS Institute, Cary, NC, USA).

## Results

### Baseline patients’ characteristics

**Figure 1:**
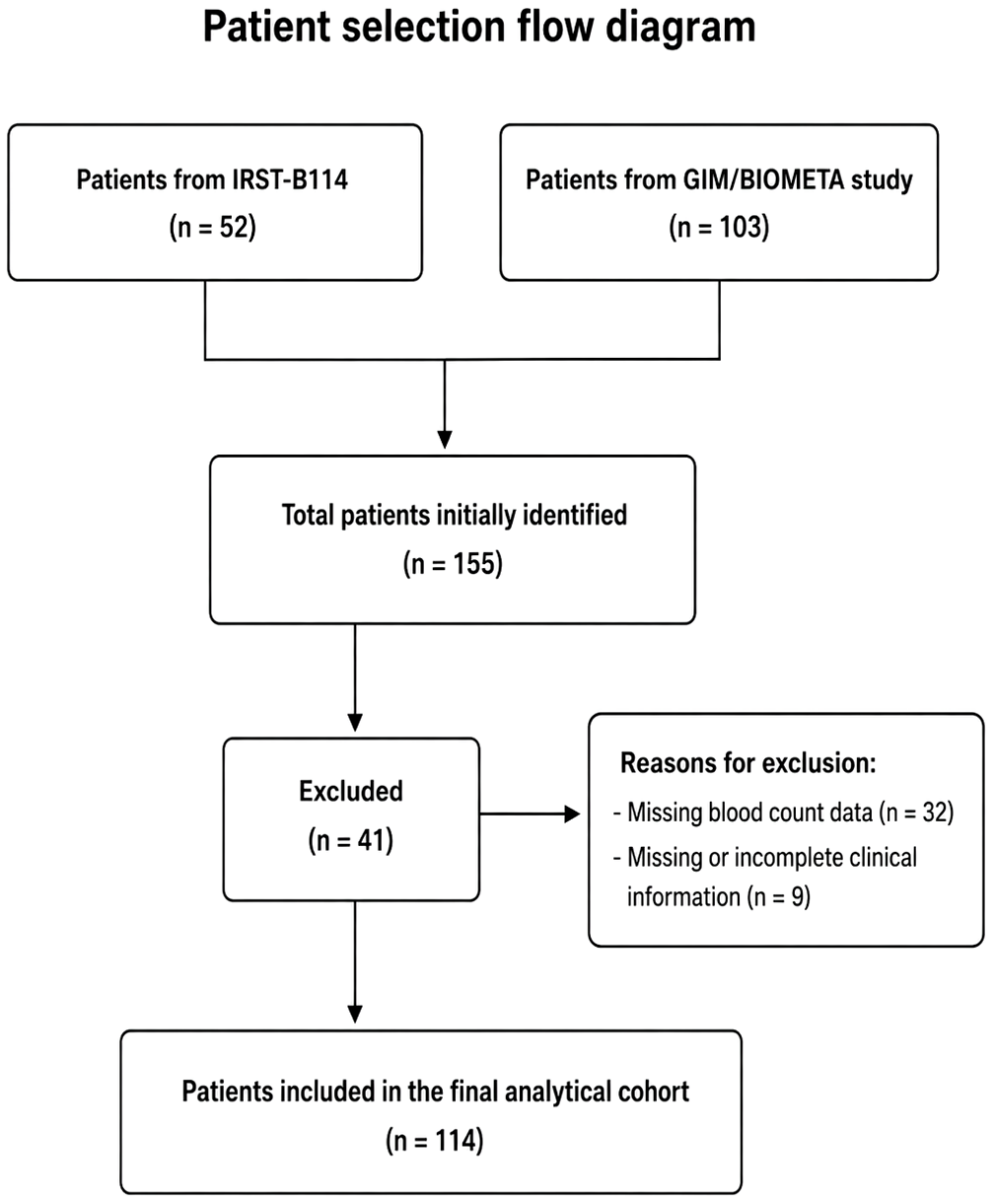
Patient selection flow diagram.

Data from 155 patients were retrospectively identified from the two parent cohorts: 103 from GIM14/BIOMETA and 52 from IRST B114. Overall, 41 patients were excluded because of missing blood count data (n = 32) or missing/incomplete clinical information (n = 9), resulting in a final analytical cohort of 114 patients (**Figure 1**).

The median age at diagnosis was 55 years (IQR 47–68). Of these, 84 patients received neoadjuvant or adjuvant treatment after the diagnosis of primary disease. Detailed first-line treatment information was available only for a subset of patients. Among patients with available treatment data, most received chemotherapy alone, while only a limited number received chemoimmunotherapy (n = 8). Three patients with PD-L1 positivity did not receive immunotherapy for clinical reasons. PD-L1 status was collected when available and assessed locally according to the assays and scoring systems used in routine clinical practice at each participating center during the study period. Because PD-L1 testing was not uniformly performed and the assay, scoring system, and specimen type were not predefined variables in the parent datasets, PD-L1 status was reported descriptively and was not included in the main multivariable models.

Other characteristics of the evaluated patients are described in **Table 1**. To further explore potential baseline imbalances according to inflammatory status, patients’ characteristics were also described according to low- versus high-NLR, PLR, SII, and MLR groups. This descriptive analysis suggested that patients with higher inflammatory indexes tended to have features of greater disease burden, including more frequent visceral involvement, liver involvement, brain metastases, and a higher number of metastatic sites. These data are reported in **Supplementary Table 1**.

**Table 1:** Patients’ characteristics.

Variable	N (%)
Age (years): median value (range, IQR)	55 (33–86, 47–68)
<55	54 (47.4)
≥55	60 (52.6)
Histology	
Ductal	83 (81.4)
Lobular	8 (7.8)
Other	11 (10.8)
Unknown	12
T at first diagnosis	
1	11 (17.5)
2	34 (54.0)
3	14 (22.2)
4	4 (6.3)
Unknown	51
T (mm): median value (IQR)	27 (19–40)
Relapsed	46 (80.7)
*De novo* metastatic disease	11 (19.3)
Unknown	57
Ki67 (%) at first diagnosis: median value (IQR)	45 (30–70)
HER2 IHC expression at first diagnosis	
0	49 (74.2)
1+	11 (16.7)
2+	5 (7.6)
3+	1 (1.5)
Unknown	48
BRCA status	
WT	36 (34.6)
Mutated	8 (7.7)
Not performed	60 (57.7)
Missing	10
PD-L1 status	
<1%	23 (28.0)
≥1%	11 (13.4)
Not performed	48 (58.6)
Unknown	32
Neoadjuvant chemotherapy	84 (74.0)
Metastatic sites at diagnosis of metastatic TNBC	
Breast	33 (29.0)
Bone	39 (34.2)
Lung	42 (36.8)
Liver	23 (20.2)
Lymph nodes	65 (57.0)
Brain	15 (13.2)
Other	26 (34.2)
Number of metastatic sites at diagnosis of metastatic TNBC	
1	41 (36.0)
2	36 (31.6)
3	19 (16.6)
≥4	18 (15.8)
Visceral involvement	
No	51 (44.7)
Yes	63 (55.3)
Ki67 status of metastasis (%): median value (IQR)	60 (35-75)
HER2 IHC expression on metastatic sites	
0	42 (62.7)
1+	16 (23.9)
2+	9 (13.4)
Unknown	47

IQR, interquartile range.

### Univariate analysis of predictors of survival

At the data cutoff, 98 PFS events and 72 OS events had occurred among the 114 included patients. At a median follow-up of 28 months (IQR 18–53), median PFS (mPFS) in the whole patient population was 8.6 months (95% CI 6.5–9.6).

High baseline NLR (≥3) significantly correlated with lower PFS: mPFS was 10.7 months (95% CI 9.4–13) in patients with NLR <3 and 4.7 months (95% CI 3.4–7.1) in those with NLR ≥3 (HR 2.76, 95% CI 1.81–4.20; p <0.0001). Regarding PLR, mPFS was better in patients with baseline PLR <210 than in patients with PLR ≥210 (9.7 versus 4.0 months, HR 2.47, 95% CI 1.58–3.85; p <0.0001). A higher MLR (≥0.34) was significantly associated with a lower mPFS than an MLR <0.34 (5.2 vs. 10.7 months, respectively; HR 2.22, 95% CI 1.46–3.37; p = 0.0002).

**Figure 2:**
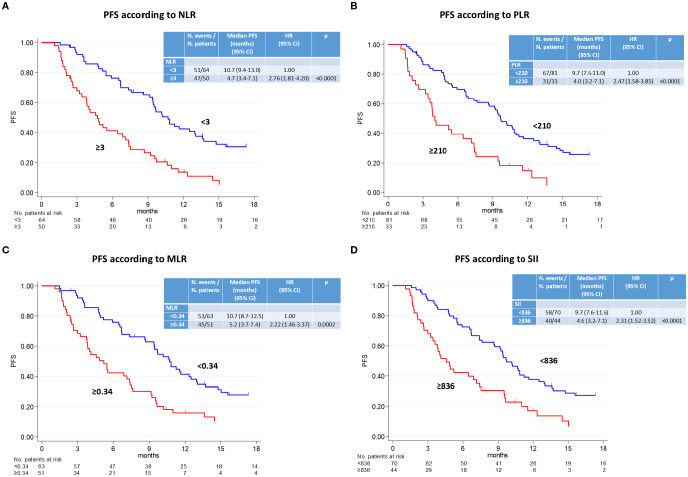
Progression free survival according to inflammatory indexes. Kaplan–Meier curves showing progression-free survival (PFS) according to baseline inflammatory index categories in patients with advanced triple-negative breast cancer: **(A)** neutrophil-to-lymphocyte ratio (NLR <3 vs ≥3); **(B)** platelet-to-lymphocyte ratio (PLR <210 vs ≥210); **(C)** monocyte-to-lymphocyte ratio (MLR <0.34 vs ≥0.34); and **(D)** systemic immune–inflammation index (SII <836 vs ≥836). High inflammatory indexes were significantly associated with shorter PFS across all evaluated biomarkers.

Similarly, an increased SII (≥836) was significantly related to a worse PFS (HR 2.31, 95% CI 1.52–3.52; p <0.0001) (**Figure 2**).

**Figure 3:**
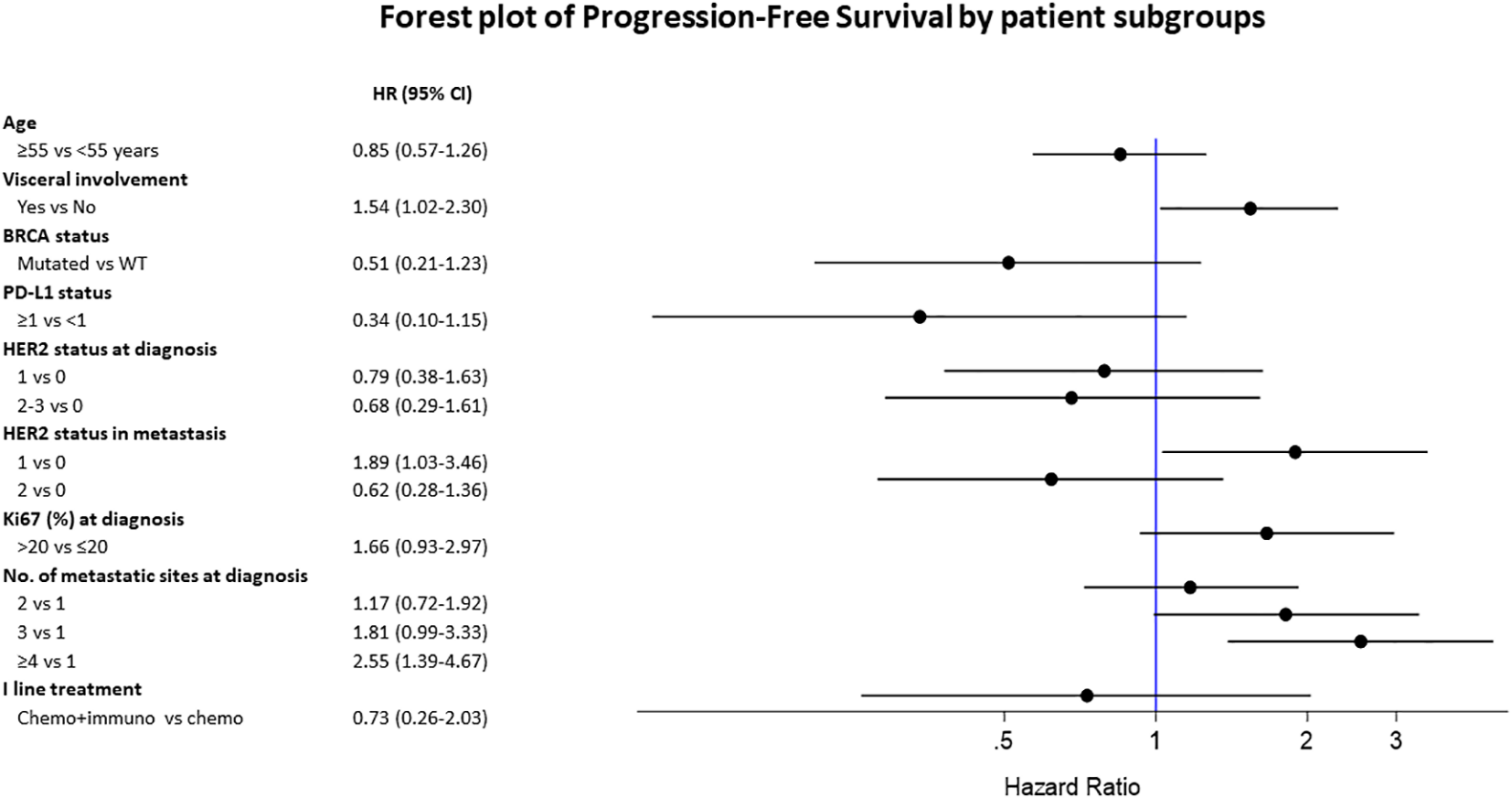
Forest plot of progression free survival by patients’ subgroups (univariable analysis).

At univariable analysis, the presence of visceral disease involvement and the number of metastatic sites were significantly associated with an increased risk of disease progression (**Figure 3**).

**Figure 4:**
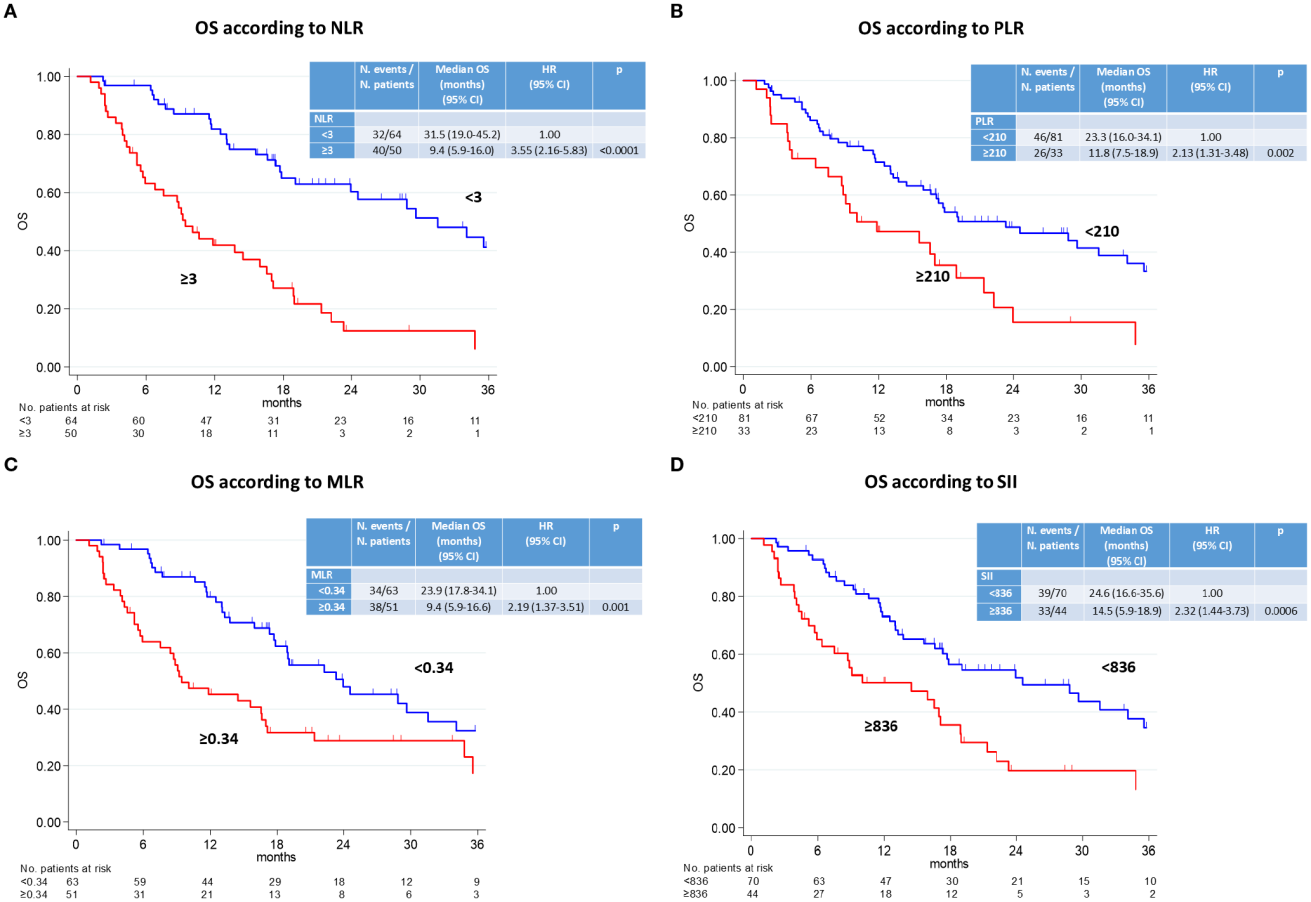
Overall survival according to inflammatory indexes. Kaplan–Meier curves showing overall survival (OS) according to baseline inflammatory index categories in patients with advanced triple-negative breast cancer: **(A)** neutrophil-to-lymphocyte ratio (NLR <3 vs ≥3); **(B)** platelet-to-lymphocyte ratio (PLR <210 vs ≥210); **(C)** monocyte-to-lymphocyte ratio (MLR <0.34 vs ≥0.34); and **(D)** systemic immune–inflammation index (SII <836 vs ≥836). Higher inflammatory indexes were associated with significantly shorter OS, supporting their prognostic role in this patient population.

Similar associations were observed for OS. Median OS (mOS) in the entire population was 17.7 months (95% CI 13.7–23.9). The mOS was significantly longer in patients with NLR <3 than in patients with NLR ≥3 (31.5 vs. 9.4 months, respectively; HR 3.55, 95% CI 2.16–5.83; p <0.0001). PLR <210 was significantly associated with a better OS (23.3 vs. 11.8 months for patients with PLR ≥210; HR 2.13, 95% CI 1.31–3.48, p = 0.002). Regarding MLR, a similar pattern was observed. MLR ≥0.34 was associated with shorter OS (9.4 vs. 23.9 months) (HR 2.19, 95% CI 1.37–3.51, p = 0.001). Finally, SII ≥836 was significantly associated with worse survival (HR 2.32, 95% CI 1.31–3.48; p = 0.0006) (**Figure 4**).

**Figure 5:**
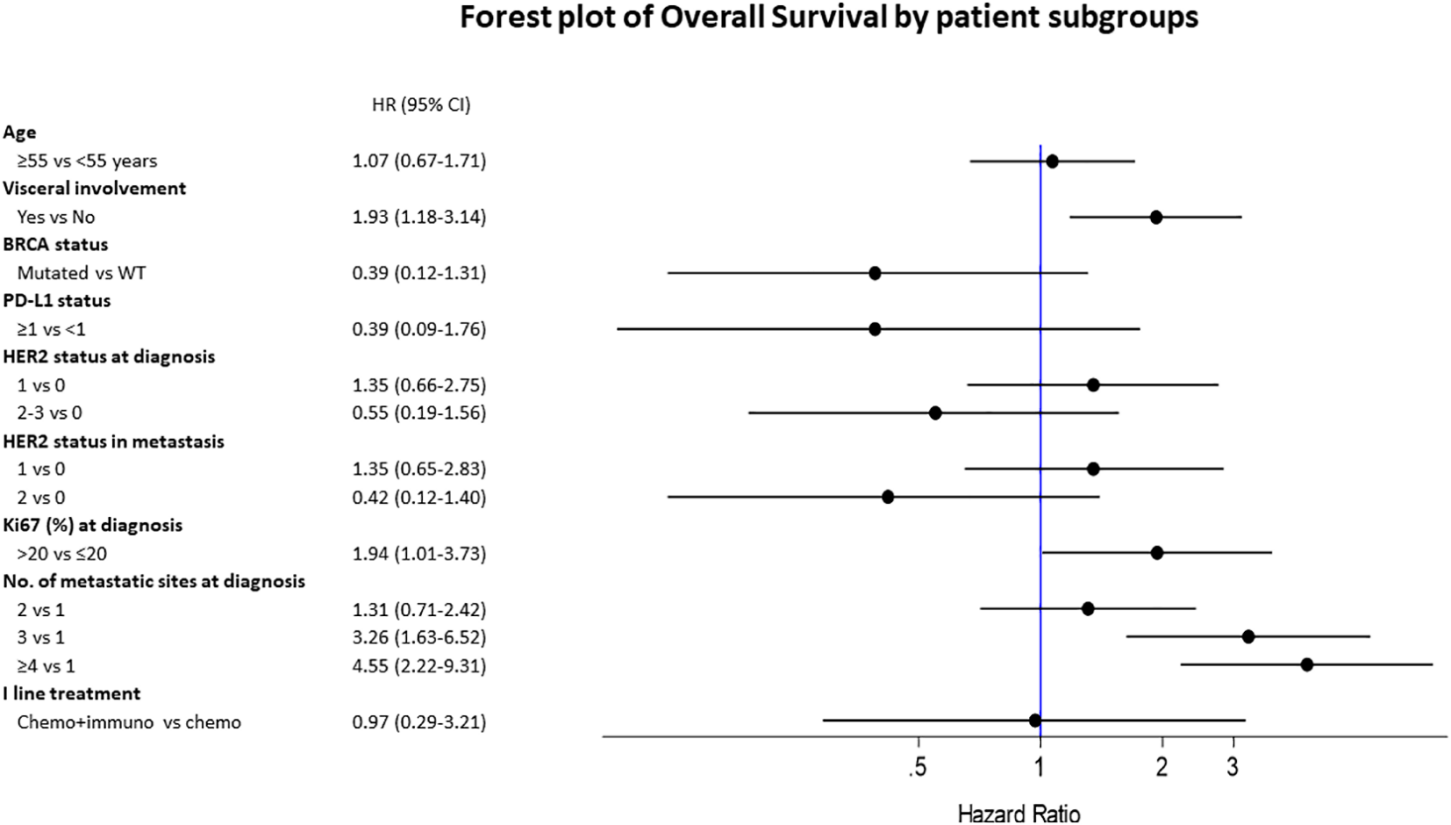
Forest plot of overall survival by patient subgroups (univariable analysis).

At univariable analysi s, the number of metastatic sites and the presence of visceral disease, as well asKi67, were significantly associated with OS (**Figure 5**).

### Sensitivity analysis of inflammatory indexes as continuous variables

To assess whether the association between inflammatory indexes and survival outcomes was preserved without relying exclusively on pre-specified cut-off values, NLR, PLR, SII, and MLR were also evaluated as log-transformed continuous variables in univariable Cox regression models. In this sensitivity analysis, logNLR, logSII, and logMLR were significantly associated with both PFS and OS, supporting the robustness of the prognostic association observed in the dichotomized analyses. Conversely, logPLR was not significantly associated with either PFS or OS when analyzed as a continuous variable, although PLR remained significantly associated with survival outcomes when assessed according to the pre-specified cut-off. These results are reported in **Supplementary Table 2**.

### Multivariate analysis of predictors of survival

Multivariable analysis including inflammatory indexes as covariates showed that high inflammatory indexes were independently associated with poor PFS. To reduce the risk of overfitting, separate parsimonious multivariable Cox regression models were built for each inflammatory index. Each model included one inflammatory index at a time and was adjusted for age, visceral involvement, and the number of metastatic sites before first-line treatment.

In these adjusted models, high NLR, PLR, MLR, and SII remained independently associated with shorter PFS. In particular, NLR ≥3 was associated with worse PFS (HR 2.40, 95% CI 1.55–3.73; p <0.0001), PLR ≥210 with worse PFS (HR 2.00, 95% CI 1.24–3.23; p = 0.005), MLR ≥0.34 with worse PFS (HR 2.09, 95% CI 1.34–3.27; p = 0.001), and SII ≥836 with worse PFS (HR 1.94, 95% CI 1.25–3.02; p = 0.003).

Regarding OS, high NLR, MLR, and SII remained independently associated with worse survival. NLR ≥3 (HR 3.03, 95% CI 1.81–5.09; p <0.0001), MLR ≥0.34 (HR 2.18, 95% CI 1.27–3.74; p = 0.005), and SII ≥836 (HR 1.92, 95% CI 1.17–3.15; p = 0.010) were associated with shorter OS. Conversely, PLR ≥210 was not significantly associated with OS in the adjusted model (HR 1.44, 95% CI 0.84–2.47; p = 0.182). The number of metastatic sites before first-line treatment remained significantly associated with OS across all models. No evidence of a violation of the proportional hazards assumption was observed for the covariates included in the Cox regression models, as none of the covariate-by-log(time) interaction terms were statistically significant (all p >0.05).

## Discussion

The study’s findings confirm that immune inflammatory indexes have significant prognostic value in metastatic TNBC. NLR, MLR, PLR, and SII were associated with lower PFS and OS, in line with the observations of other authors (13). The study’s focus on immune inflammatory indexes provides insights into the functional status of the immune system and its impact on TNBC progression, contributing to the growing body of evidence on the interplay between immunity and cancer and underlining the potential influence of immune dysregulation on disease trajectory.

To address the potential loss of information related to dichotomization, we also evaluated inflammatory indexes as log-transformed continuous variables. In this sensitivity analysis, logNLR, logSII, and logMLR remained significantly associated with both PFS and OS, supporting the robustness of their prognostic value beyond the use of pre-specified thresholds. Conversely, logPLR was not significantly associated with either PFS or OS when analyzed as a continuous variable, although PLR retained prognostic value when assessed using the pre-specified cut-off. This finding suggests that the prognostic relevance of PLR may be more dependent on threshold-based categorization and should therefore be interpreted with greater caution.

In particular, relative lymphopenia seems to contribute considerably to the mechanism of tumor immune escape (14). The tumor microenvironment presents a state of persistent chronic inflammation that is particularly important for tumor progression and growth, promoting the recruitment of regulatory T cells (Tregs), tumor-associated macrophages and myeloid-derived suppressor cells. The chemokine and interleukin milieu promoted by the tumor microenvironment contributes to the systematic inflammatory response (12). It seems that inflammatory cells, such as monocytes, play an important role as chaperones for circulating tumor cells during their dissemination to new metastatic niches (11, 15). These indexes could serve as accessible and cost-effective tools to support prognostic stratification in metastatic TNBC.

Moreover, a previous study suggested a possible association between NLR and treatment outcomes, highlighting its potential clinical relevance (16).

Patients with absent or low hormone receptor (HR) expression (<10%) were included in the analysis, as HR-low tumors are known to be biologically similar to classical triple-negative disease in terms of prognosis and response to the same treatments (17).

Regarding the expression of HER2, although data were available for only a subset of patients, some considerations can still be made.

The HER2-low condition is becoming increasingly important, and an attempt is underway to categorize this patient population as a true subgroup, challenging the traditional subdivision of biological subtypes of breast cancer. However, retrospective data on this new category seem to suggest that HER2 expression in localized triple-negative breast cancer does not affect prognosis (18–20). In contrast, conflicting data are emerging in the metastatic setting instead, suggesting that HER2-low expression may be associated with slightly improved OS in HR-negative advanced tumors and the availability of antibody–drug conjugates is changing the treatment landscape (21).

It is important to consider also that HER2 IHC scores can be highly variable, depending on the timing and site of the biopsy and the stage of the disease course (22). Our data do not show any strong correlation between HER2 IHC expression and immune inflammatory indexes, and the results are inconclusive. Therefore, further studies are needed to clarify this relationship.

Our study exhibits several strengths that bolster the credibility of its findings. The use of a multicenter observational design involving data from the GIM14 experience adds to the study’s robustness. Moreover, the use of pre-specified cut-off values derived from a previously published study by our group enhances the reproducibility of the analysis and minimizes the risk of overfitting associated with data-driven threshold selection, while allowing us to assess the prognostic relevance of these thresholds in an advanced/metastatic TNBC cohort. Nevertheless, formal external validation in an independent cohort is still required.

Despite its strengths, our study has certain limitations that warrant consideration. The retrospective–prospective design, the relatively limited cohort size (114 patients), and the local assessment of biological characteristics, including hormone receptor expression, may have affected the generalizability of the findings. In particular, eligibility was based on TNBC status in the advanced/metastatic setting and allowed inclusion of tumors with low ER and/or PR expression. Although hormone receptor–low/HER2-negative tumors are often clinically managed similarly to classical TNBC, the applicability of our results should be interpreted with caution. The interpretation of the results is also limited by missing data for several relevant clinicopathological and biomarker variables. Our analysis focused on patients who received first-line systemic treatment, thereby excluding untreated patients, who might have a worse outcome.

The descriptive comparison of baseline characteristics according to inflammatory-index groups showed that patients with higher inflammatory indexes tended to present with features of greater disease burden, including visceral involvement and a higher number of metastatic sites. Although multivariable models were adjusted for visceral involvement and the number of metastatic sites, which were considered clinically relevant measures of disease burden, residual confounding cannot be excluded. This aspect should be considered when interpreting the prognostic association between inflammatory indexes and survival outcomes.

Another relevant limitation is that peripheral inflammatory indexes may be influenced by non-cancer-related factors. Detailed information on performance status, intercurrent infections, recent corticosteroid exposure, hospitalization, granulocyte colony-stimulating factor use related to previous treatments, recent surgery, and chronic inflammatory comorbidities at the time of blood sampling was not systematically available in the datasets and could not be included in the adjusted models. Therefore, residual confounding related to unmeasured inflammatory or clinical conditions cannot be excluded.

The patients included in the cohort were predominantly treated with first-line chemotherapy, with only a small subset receiving immunotherapy. This reflects the treatment landscape during the study period but limits the direct applicability of our findings to current first-line metastatic TNBC practice. In addition, PD-L1 testing was not uniformly performed, and the assay, scoring system, and specimen type were not systematically recorded. Therefore, treatment type and PD-L1 status could not be systematically included in the adjusted models. The prognostic value of inflammatory indexes should be further validated in contemporary metastatic TNBC cohorts treated with chemoimmunotherapy and, increasingly, antibody–drug conjugates in the advanced/metastatic setting (23–25).

Although the use of cut-off values from a prior study supports reproducibility, these thresholds may not fully capture the prognostic complexity of the current patient population. However, the sensitivity analysis using log-transformed continuous variables confirmed the prognostic relevance of NLR, SII, and MLR, partially addressing this limitation. The study’s reliance on immune inflammatory indexes alone might overlook other relevant factors influencing TNBC progression and outcomes, such as genetic mutations or specific immune cell populations. Further investigations are needed to explore potential synergies between immune indexes and other biomarkers.

This study opens avenues for future research in multiple directions. Larger, prospective studies with various patient populations could validate and refine these findings. Exploring the molecular mechanisms underlying the observed associations between immune indexes and clinical outcomes could provide deeper insights into TNBC pathogenesis. Furthermore, investigating the potential of combining immune inflammatory indexes with other biomarkers or genetic profiling might enhance the accuracy of prognostication. Future studies integrating inflammatory indexes with clinical, pathological, and molecular biomarkers may improve prognostic stratification in metastatic TNBC.

In conclusion, NLR, PLR, SII, and MLR were associated with survival outcomes in advanced/metastatic TNBC. Although our results require validation in larger prospective studies and in contemporary cohorts treated with chemoimmunotherapy and novel agents, inflammatory ratios may represent feasible and routinely available prognostic biomarkers in metastatic TNBC.

## Data Availability

The dataset used and analyzed during the current study is available from the corresponding author upon reasonable request. Requests to access the datasets should be directed to emanuela.scarpi@irst.emr.it.
