# Supplementary material for: Inflammatory indexes as prognostic biomarkers in advanced triple negative breast cancer patients
Source: Front Oncol. 2026 Jul 15;16:1876224. doi: 10.3389/fonc.2026.1876224 (PMC13414733; doi:10.3389/fonc.2026.1876224)
Supplement: Supplementary file 1 [file Table1.docx]

**Table 1 Supplementary.** Univariable analysis of PFS and OS with log-transformed inflammatory indexes as continuous variables.

|  | **PFS** | | | **OS** | | |
| --- | --- | --- | --- | --- | --- | --- |
|  | **HR (95% CI)** | **p** | **HR (95% CI)** | | **p** |  |
| **Age** | 0.99 (0.97-1.01) | 0.281 | 1.01 (0.98-1.02) | | 0.954 |  |
| **logNLR** | 1.71 (1.20-2.42) | 0.003 | 2.12 (1.49-3.02) | | <0.0001 |  |
| **logPLR** | 1.08 (0.80-1.46) | 0.626 | 1.28 (0.87-1.88) | | 0.213 |  |
| **logSII** | 1.43 (1.08-1.90) | 0.012 | 1.69 (1.27-2.24) | | 0.0003 |  |
| **logMLR** | 1.22 (1.01-1.46) | 0.035 | 1.31 (1.09-1.57) | | 0.004 |  |

**Table 2 Supplementary.** Patients’ characteristics according to inflammation-index group

|  | **NLR** | | **PLR** | | **SII** | | **MLR** | |
| --- | --- | --- | --- | --- | --- | --- | --- | --- |
|  | **<3** | **≥3** | **<210** | **≥210** | **<836** | **≥836** | **<0.34** | **≥0.34** |
|  | **N (%)** | **N (%)** | **N (%)** | **N (%)** | **N (%)** | **N (%)** | **N (%)** | **N (%)** |
| **Age (years):** median value (range, IQR) | 55 (34-86, 47-69) | 56 (33-83, 47-67) | 58 (33-86, 48-71) | 52 (36-78, 47-61) | 55 (33-86, 47-69) | 56 (36-83, 48-66) | 55 (34-86, 47-66) | 57 (33-83, 48-69) |
| <55 | 31 (48.4) | 23 (46.0) | 35 (43.2) | 19 (57.6) | 33 (47.1) | 21 (47.7) | 31 (49.2) | 23 (45.1) |
| ≥55 | 33 (51.6) | 27 (54.0) | 46 (56.8) | 14 (42.4) | 37 (52.9) | 23 (52.3) | 32 (50.8) | 28 (54.9) |
| **Istology** |  |  |  |  |  |  |  |  |
| Ductal | 47 (78.3) | 36 (85.7) | 59 (80.8) | 24 (82.8) | 51 (78.5) | 32 (86.5) | 48 (84.2) | 35 (77.8) |
| Lobular | 6 (10.0) | 2 (4.8) | 6 (8.2) | 2 (6.9) | 6 (9.2) | 2 (5.4) | 4 (7.0) | 4 (8.9) |
| Other | 7 (11.7) | 4 (9.5) | 8 (11.0) | 3 (10.3) | 8 (12.3) | 3 (8.1) | 5 (8.8) | 6 (13.3) |
| Unknown | 4 | 8 | 8 | 4 | 5 | 7 | 6 | 6 |
| **T at first diagnosis** |  |  |  |  |  |  |  |  |
| 1 | 3 (8.8) | 8 (27.6) | 7 (17.1) | 4 (18.2) | 4 (10.8) | 7 (26.9) | 4 (11.8) | 7 (24.2) |
| 2 | 21 (61.8) | 13 (44.8) | 24 (58.6) | 10 (45.5) | 21 (56.8) | 13 (50.0) | 21 (61.8) | 13 (44.8) |
| 3 | 9 (26.5) | 5 (17.3) | 8 (19.5) | 6 (27.3) | 10 (27.0) | 4 (15.4) | 8 (23.5) | 6 (20.7) |
| 4 | 1 (2.9) | 3 (10.3) | 2 (4.9) | 2 (9.1) | 2 (5.4) | 2 (7.7) | 1 (2.9) | 3 (10.3) |
| Unknown | 30 | 21 | 40 | 11 | 33 | 18 | 29 | 22 |
| **T (mm):** median value (IQR) | 25 (20-37) | 28 (17-44) | 25 (18-37) | 30 (21-55) | 26 (20-38) | 28 (18-40) | 25 (21-38) | 28 (18-40) |
| **Relapsed** | 28 (80.0) | 18 (81.8) | 34 (82.9) | 12 (75.0) | 32 (80.0) | 14 (82.3) | 23 (79.3) | 23 (82.1) |
| **De novo metastatic disease** | 7 (20.0) | 4 (18.2) | 7 (17.1) | 4 (25.0) | 8 (20.0) | 3 (17.7) | 6 (20.7) | 5 (17.9) |
| **Unknown** | 28 | 29 | 40 | 17 | 30 | 17 | 34 | 23 |
| **Ki67 (%) at first diagnosis:**  median value (IQR) | 40 (25-65) | 60 (35-70) | 40 825-65) | 65 (40-75) | 40 (25-65) | 60 (35-70) | 40 (30-70) | 53 (28-70) |
| **HER2 ICH expression at first diagnosis** |  |  |  |  |  |  |  |  |
| 0 | 19 (70.4) | 30 (76.9) | 29 (74.4) | 20 (74.1) | 26 (74.3) | 23 (74.2) | 18 (62.1) | 31 (83.8) |
| 1+ | 5 (18.5) | 6 (15.4) | 6 (15.4) | 5 (18.5) | 6 (17.1) | 5 (16.1) | 6 (20.7) | 5 (13.5) |
| 2+ | 2 (7.4) | 3 (7.7) | 3 (7.7) | 2 (7.4) | 2 (5.7) | 3 (9.7) | 4 (13.8) | 1 (2.7) |
| 3+ | 1 (3.7) | 0 | 1 (2.6) | 0 | 1 (2.9) | 0 | 1 (3.4) | 0 |
| Unknown | 37 | 11 | 42 | 6 | 35 | 13 | 34 | 14 |
| **BRCA status** |  |  |  |  |  |  |  |  |
| WT | 17 (29.3) | 19 (41.3) | 22 (30.2) | 14 (45.2) | 19 (29.2) | 17 (43.6) | 19 (34.5) | 17 (34.7) |
| Mutated | 5 (8.6) | 3 (6.5) | 5 (6.8) | 3 (9.6) | 6 (9.2) | 2 (5.1) | 4 (7.3) | 4 (8.2) |
| Not performed | 36 (62.1) | 24 (52.2) | 46 (63.0) | 14 (45.2) | 40 (61.6) | 20 (51.3) | 32 (58.2) | 28 (57.1) |
| Missing | 6 | 4 | 8 | 2 | 5 | 5 | 8 | 2 |
| **PD-L1 status** |  |  |  |  |  |  |  |  |
| <1% | 10 (20.8) | 13 (38.2) | 16 (28.1) | 7 (28.0) | 13 (25.0) | 10 (33.3) | 15 (33.3) | 8 (21.6) |
| ≥1% | 6 (12.5) | 5 (14.7) | 6 (10.5) | 5 (20.0) | 6 (11.5) | 5 (16.7) | 6 (13.4) | 5 (13.5) |
| Not performed | 32 (66.7) | 16 (47.1) | 35 (61.4) | 13 (52.0) | 33 (63.5) | 15 (50.0) | 24 (53.3) | 24 (64.9) |
| Unknown | 16 | 16 | 24 | 8 | 18 | 14 | 18 | 14 |
| **Neoadjuvant chemotherapy** | 48 (84.2) | 36 (83.7) | 61 (85.9) | 23 (79.3) | 53 (82.8) | 31 (86.1) | 47 (85.4) | 37 (82.2) |
| **Metastatic sites at diagnosis of metastatic TNBC** |  |  |  |  |  |  |  |  |
| Breast | 21 (32.8) | 12 (24.0) | 21 (25.9) | 12 (36.4) | 21 (70.0) | 12 (27.3) | 21 (33.3) | 12 (23.5) |
| Bone | 22 (34.4) | 17 (34.0) | 27 (33.3) | 12 (36.4) | 27 (38.6) | 12 (27.3) | 22 (34.9) | 17 (33.3) |
| Lung | 19 (29.6) | 23 (46.0) | 27 (33.3) | 15 (45.4) | 25 (35.7) | 17 (38.6) | 19 (30.2) | 23 (45.1) |
| Liver | 8 (12.5) | 15 (30.0) | 16 (19.7) | 7 (21.2) | 7 (10.0) | 16 (36.4) | 6 (9.5) | 17 (33.3) |
| Lymphnodes | 32 (50.0) | 33 (66.0) | 42 (51.8) | 23 (69.7) | 36 (51.4) | 29 (65.9) | 36 (57.1) | 29 (56.9) |
| Brain | 5 (7.8) | 10 (20.0) | 7 (8.6) | 8 (24.2) | 6 (8.6) | 9 (20.4) | 5 (7.9) | 10 (19.6) |
| Other | 14 (37.8) | 12 (30.8) | 15 (30.0) | 11 (42.3) | 14 (34.1) | 12 (34.3) | 15 (36.6) | 11 (31.4) |
| **Number of metastatic sites at diagnosis of metastatic TNBC** |  |  |  |  |  |  |  |  |
| 1 | 25 (39.1) | 16 (32.0) | 32 (39.5) | 9 (27.3) | 28 (40.0) | 13 (29.6) | 22 (34.9) | 19 (37.3) |
| 2 | 26 (40.6) | 10 (20.0) | 30 (37.0) | 6 (18.2) | 27 (38.6) | 9 (20.4) | 25 (39.7) | 11 (21.6) |
| 3 | 7 (10.9) | 12 (24.0) | 10 (12.3) | 9 (27.3) | 8 (11.4) | 11 (25.0) | 10 (15.9) | 9 (17.6) |
| ≥4 | 6 (9.4) | 12 (24.0) | 9 (11.1) | 9 (27.3) | 7 (10.0) | 11 (25.0) | 6 (9.5) | 12 (23.5) |
| **Visceral involvement** |  |  |  |  |  |  |  |  |
| No | 36 (56.2) | 15 (30.0) | 39 (48.1) | 12 (36.4) | 37 (52.9) | 14 (31.8) | 38 (60.3) | 13 (25.5) |
| Yes | 28 (43.8) | 35 (70.0) | 42 (51.9) | 21 (63.6) | 33 (47.1) | 30 (68.2) | 25 (39.7) | 38 (74.5) |
| **Ki67 status of metastasis (%):**  median value (IQR) | 50 (25-73) | 70 (47-75) | 50 (23-74) | 70 (65-80) | 52 (25-73) | 70 (35-75) | 50 (25-73) | 70 (35-75) |
| **HER2 IHC expression on metastatic sites** |  |  |  |  |  |  |  |  |
| 0 | 28 (71.8) | 14 (50.0) | 32 (65.3) | 10 (55.6) | 27 (69.2) | 15 (53.6) | 27 (62.8) | 15 (62.5) |
| 1+ | 6 (15.4) | 10 (35.7) | 9 (18.4) | 7 (38.9) | 7 (18.0) | 9 (32.1) | 9 (20.9) | 7 (29.2) |
| 2+ | 5 (12.8) | 4 (14.3) | 8 (16.3) | 1 (5.6) | 5 (12.8) | 4 (14.3) | 7 (16.3) | 2 (8.3) |
| Unknown | 25 | 22 | 32 | 15 | 31 | 16 | 20 | 27 |
